# ThermoCyte: an inexpensive open-source temperature control system for *in vitro* live-cell imaging

**DOI:** 10.1098/rsos.231037

**Published:** 2023-11-29

**Authors:** Ross O'Carroll, James P. Reynolds, Mazen Al-Roqi, Emmanuelle Damilola Aiyegbusi, Dearbhaile Dooley

**Affiliations:** ^1^ School of Medicine, Health Sciences Centre, University College Dublin, Belfield, Dublin D04 V1W8, Ireland; ^2^ Conway Institute of Biomolecular & Biomedical Research, University College Dublin, Belfield, Dublin D04 V1W8, Ireland

**Keywords:** *in vitro*, live-cell imaging, open-source, temperature control, cell biology, inexpensive

## Abstract

Live-cell imaging is a common technique in microscopy to investigate dynamic cellular behaviour and permits the accurate and relevant analysis of a wide range of cellular and tissue parameters, such as motility, cell division, wound healing responses and calcium (Ca^2^^+^) signalling in cell lines, primary cell cultures and *ex vivo* preparations. Furthermore, this can occur under many experimental conditions, making live-cell imaging indispensable for biological research. Systems which maintain cells at physiological conditions outside of a CO_2_ incubator are often bulky, expensive and use proprietary components. Here we present an inexpensive, open-source temperature control system for *in vitro* live-cell imaging. Our system ‘ThermoCyte’, which is constructed from standard electronic components, enables precise tuning, control and logging of a temperature ‘set point’ for imaging cells at physiological temperature. We achieved stable thermal dynamics, with reliable temperature cycling and a standard deviation of 0.42°C over 1 h. Furthermore, the device is modular in nature and is adaptable to the researcher's specific needs. This represents simple, inexpensive and reliable tool for laboratories to carry out custom live-cell imaging protocols, on a standard laboratory bench, at physiological temperature.

## 1. Introduction

Live-cell imaging is a powerful tool for investigating cellular behaviour in many disease models [1–4]. In most cases, this method uses time-lapse microscopy to acquire a series of images, which are then analysed computationally. Many different optical microscopy techniques exist, including brightfield, phase-contrast, differential interference contrast (DIC) and fluorescence microscopy [5–8]. Although there are many differences to these techniques and the protocols that accompany them, they share a common requirement: live cells should be imaged as close to physiological conditions as possible [9]. This is critical to maximize the relevance of the imaging study and to improve reliability of results obtained. To satisfy this criterion, microscopists often use commercially available live-cell microscopes, as part of shared core facilities. These microscopes may have environmental control chambers included, which appropriately stabilize temperature, CO_2_ concentration and humidity for the duration of imaging. Although effective, these systems are large and often require dedicated imaging space. Additionally, they are expensive and regularly use bespoke proprietary components, limiting future adaptation without specialist input.

To more easily facilitate live-cell imaging at physiological conditions, many commercial solutions have been developed by a number of laboratory hardware developers. These include (but are not limited to) in-line heaters, stage-top incubators and integrated imaging systems with built-in environmental controls. These devices are similarly effective at controlling environmental variables such as CO_2_ concentration, temperature and humidity for a variety of imaging studies. However, they remain expensive options for researchers, especially those who may be new to live-cell imaging and do not want to over-invest at the outset. Further, these solutions are arguably less adaptable without proprietary hardware, and this may be a disadvantage for those who have not fully optimized their imaging protocols before purchase [10–12]. Thus, we have identified a need for an inexpensive but effective tool to image cells in real time at physiological conditions.

To address this, we have developed an inexpensive, open-source temperature control module for use with peristaltic and gravity-assisted flow circuits. ‘ThermoCyte’, our custom live-cell heating device, is small, uses standard electronic components, open-source Arduino microcontrollers, and importantly, operates directly on the microscope stage with low footprint. Using the design described below, we have achieved stable temperature control in a variety of conditions. We have tested several protocols in our system, the most relevant of which we are using to investigate the role of microglia in *in vitro* models of neuroinflammation [13–15]. Behavioural analysis of microglia using imaging studies is a well-established technique in the field of neuroinflammation [16,17]. We extensively validated our temperature control system using BV2 microglial calcium-imaging, and are confident that it represents an effective, inexpensive option for research groups in need of a cost-effective live-cell temperature control module.

## 2. Material and methods

### 2.1. Modelling

**Figure 1.**
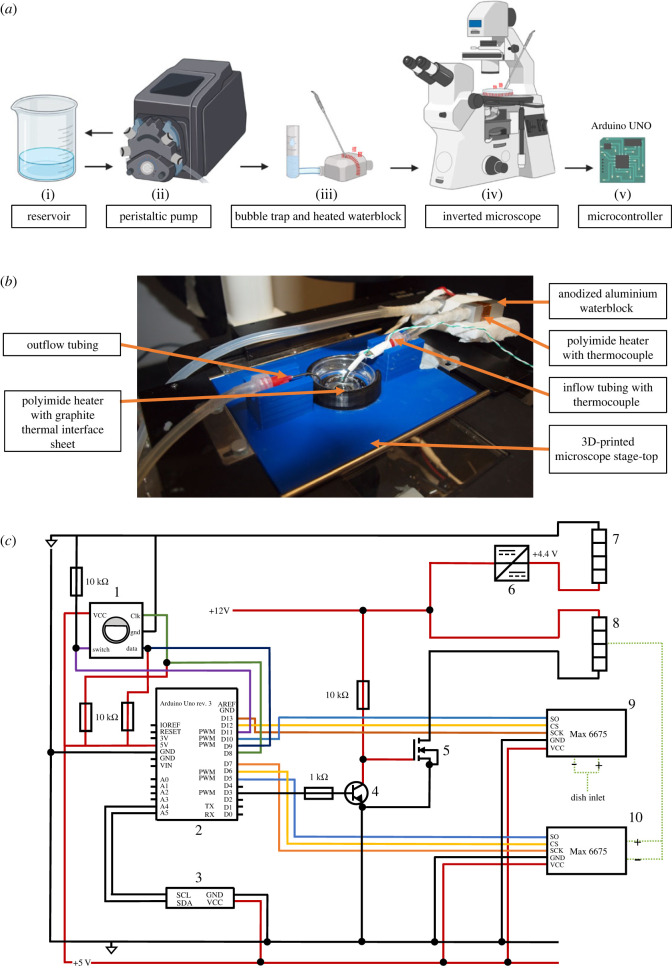
Design and configuration of heating system. (*a*) Schematic outlining individual components of heating set-up, left to right: (i) Reservoir containing artificial cerebrospinal fluid (aCSF), (ii) peristaltic pump (not controlled by Arduino) and rubber tubing, (iii) bubble trap, anodized aluminium waterblock, 12 V polyimide resistive heating strip and K-type thermocouple, (iv) custom-modified Nikon Eclipse TS100 inverted epifluorescence microscope with Petri dish, thermocouple and 12 V polyimide resistive heating strip, (v) Arduino UNO REV 3. (*b*) Photograph of stage-top heating system, showing the aluminium waterblock and associated polyimide heater, three-dimensional-printed stage-top, Petri dish heating element, and inflow/outflow tubing. (*c*) Circuit diagram of heating mechanism and integration with Arduino: (1) rotary encoder, (2) Arduino UNO REV 3, (3) I2C 16 × 2 liquid crystal display (LCD), (4) bipolar junction transistor (BJT), (5) metal oxide semiconductor field-effect transistor (MOSFET), (6) DC-DC step-down converter/voltage regulator, (7,8) 12 V polyimide resistive heating strips, (9,10) Max 6675 K-type thermocouple amplifiers. Some parts of figures created with Biorender.com.

Three-dimensional models of the stage-top Petri dish holder and cover were designed using Autodesk ‘Tinker CAD’, a free browser-based CAD software solution. Files were exported to ‘stl’ format for three-dimensional printing. Circuit diagram (figure 1*a*) was created using Microsoft Powerpoint.

### 2.2. Three-dimensional printing

A Creality Ender 3 printer was used to three-dimensionally print all polylactic acid (PLA)-based components in the system. A 0.4 mm print nozzle, tempered glass print bed and generic PLA were used. Nozzle temperature was set to 210°C, and the print bed was set to 50°C. Files were prepared and sliced using Prusa Slicer software.

### 2.3. Electronics and hardware

An Arduino UNO (cat. no. A000066, Arduino) microcontroller was used in combination with an two RS Pro K-type thermocouples (cat. no. A000066, Radionics), two MAX 6675 thermocouple amplifiers (cat. no. MAX6657, Irish Electronics), a 12 V DC-DC ‘buck’ step-down converter (model no. DFR0379, cat. no. 3769933, Farnell), a solderless breadboard (cat. no. 340-002-1, Radionics), an I2C liquid crystal display (LCD) (cat. no. SKU:DFR0063, DFRobot), jumper wires (cat. no. K000007, Arduino), a variable 5V-12 V AC adaptor (cat. no. 7023259, Radionics), two 12 V flexible polyimide strip heaters (cat. no. B076PD7TXW, Icstation, Amazon.com), a 32A/60 V N-channel metal–oxide–semiconductor field-effect transistor (model no. FQP30N06L, cat. no. 807-5863, Radionics), an NPN-type bipolar junction transistor (model no. s8050, cat. no. 808-0449, Radionics), a graphite-based thermal interface sheet (cat. no. EYG-S091210DP), a variable speed peristaltic pump (model no. MP II, cat. no. MA1 70-2027, Harvard Apparatus), and an aluminium waterblock (cat. no. GL.001.340, Gleanntronics) which was kindly clear anodized by CPS Ltd, Santry, Co. Dublin, Ireland.

### 2.4. Code

Code was written (adapted from open-source material [18]) to interface readings from the two K-type thermocouple amplifiers with the Arduino and the waterblock polyimide heating strip. In brief, a waterblock target temperature (set point) is set in the Arduino IDE, allowing the strip heater to receive full current via the proportional, integral, derivative (PID) control system. Once target temperature is reached, PID code automatically adjusts the current to the heating strip, maintaining its temperature around the set point. The strip heater around the Petri dish was connected to a constant 4.4 V via the DC-DC step-down converter. See electronic supplementary material, appendix for code.

### 2.5. BV2 microglia culture

BV2 cells were kindly gifted by Dr Costello, Conway Institute of Biomolecular and Biomedical research, University College Dublin (UCD). Cells were cultured in complete Dulbecco's Modified Eagle's Medium (cDMEM): DMEM supplemented with 10% fetal bovine serum (FBS, cat. no. 10270106, Gibco), 1% penicillin/streptomycin (cat. no. 15 070-063, Gibco) and 15 mM HEPES buffer (cat. no. H0887, Sigma) for approximately 4 days until confluent. Cultures were maintained in a humidified incubator at 37°C with 5% CO_2_. Medium was replenished every 2–3 days. At 70–90% confluency, cells up to passage 18 were harvested and used for subsequent experiments.

### 2.6. BV2 calcium imaging

Fluo-4 AM calcium-dependent dye was purchased from ThermoFisher Scientific (cat. no. F14201). BV2 cells were subcultured and counted using a haemocytometer. 10 000 cells were plated onto 0.01% poly-L-lysine (cat. no. P4707-50ML, Sigma Aldrich)-treated 35 mm glass confocal dishes (cat. no. P35G-1.5-20-C, MatTek), and incubated at 37°C for 3 days. Cells were washed three times with warmed sterile phosphate-buffered saline (PBS) (hereafter implied) and incubated with Fluo4-AM solution in Hanks' balanced salt solution (HBSS) for 1 h, 37°C. Cells were serum-starved during loading protocol for 1 h. Cells were washed and imaged at room temperature (approx. 20°C) and 37°C degrees in artificial cerebrospinal fluid (aCSF) (in mM; NaCl, 125; KCl, 2.5; NaH_2_PO_4_, 1.25; NaHCO_3_, 11.9; CaCl_2_, MgSO_4_∙7H_2_O, 1.3; D-glucose, 18; HEPES, 10) using a customized fluorescence microscope (see §2.8) and ThermoCyte temperature controller.

### 2.7. Mouse-induced pluripotent stem cell-derived microglia culture

Murine-induced pluripotent stem cell (miPSC)-derived microglia (CX_3_CR1^eGFP/+^CCR2^RFP/+^) were cultured as described previously by Quarta *et al*., [19]. miPSCs were kindly gifted by Prof. Ponsaert, University of Antwerp, Antwerp, Belgium. In brief, miPSCs were seeded on mouse irradiated embryonic fibroblasts (MEFs). Once confluent, miPSCs were separated from MEFs using trypsin and transferred to agarose-coated Petri dishes until embryoid bodies were formed. Once embryoid bodies were formed, miPSCs were transferred to gelatin-coated flasks. Supernatant, containing primitive macrophage progenitors, was collected and co-cultured with mouse neural stem cells (NSCs) derived from p13-16 mouse forebrain. After a period of 7–10 days co-culture, mature GFP^+^ microglia are detectable in the culture, and can be further cultured using complete Glasgow's Modified Eagle's Medium (cGMEM). 10 000 mature GFP^+^ microglia were seeded onto a 35 mm Petri dish coated with 20 µg ml^−1^ fibronectin, in cGMEM, and incubated at 37**°**C, 5% CO_2_ for 3 days.

### 2.8. Imaging acquisition

Images were acquired using a Nikon eclipse TS100 brightfield microscope, custom-modified to support fluorescence imaging. A 470 nm LED (cat. no. M470L5, Thorlabs) was mounted at a rear optical port and routed to the objective/camera through a FITC-filter cube, consisting of a 475/35 nm bandpass (excitation) filter (cat. no. MF475-35, Thorlabs), a 530/43 nm bandpass (emission) filter (cat. no. MF540-43, Thorlabs), a 498 nm longpass dichroic mirror (cat. no. MD498, Thorlabs) and assorted optomechanical components for mounting. Images were captured using an Optika CP-6 digital camera (cat. no. 57549) through one of three objective lenses (Nikon Plan 10X 0.25 NA, Nikon Plan Fluor 20X 0.45 NA and Nikon Plan Fluor 40X 0.60 NA).

### 2.9. Image analysis

Images were analysed using ImageJ and Python. The ImageJ plugin ‘moco’ was used to correct for cellular motion and drift [20]. The plugin ‘napari-bleach-correct’ was used with Napari image viewer (python), based on the original ImageJ plugin by Miura *et al.* [21]. The Python package ‘Cellpose’ and ‘cyto2’ algorithm were used for object detection during analysis [22,23]. Fiji (https://imagej.net/software/fiji/) was used for temporal heat map production and other image analysis. Calcium fluctuations were assessed using measures of relative changes in fluorescence intensity as a ratio versus periods of sustained low fluorescence (F_0_) indicating steady-state intracellular calcium levels (referred to as ΔF/F). A calcium transient was defined as a period of greater than 0.5 Δ*F*/*F* elapsing for at least 2 s. Python code was written for visualization, peak detection and area-under-curve (AUC) measurements.

### 2.10. Miscellaneous

*Danio rerio* larvae, Tg(mpx:GFP, mpeg1:mCherry) at 4 days post-fertilization, were kindly provided by Dr Darrell Andrews and Dr Breandán Kennedy, School of Biomolecular and Biomedical Science, University College Dublin. *Danio rerio* larvae were imaged under tricaine anaesthesia. Desiccated *Saccharomyces cerevisiae* was obtained from Tesco PLC.

### 2.11. Statistical analysis

Statistical analysis was carried out using GraphPad Prism 9.5.1 and the python package ‘statsmodels’ v. 0.13.5 (python 3.10.9). For all experiments, a 95% confidence interval was used and a *p*-value ≤ 0.05 was considered as statistically significant.

## 3. Results

### 3.1. Design and construction of a cheap, open-source temperature control system for *in vitro* live-cell imaging on the bench-top

**Figure 2.**
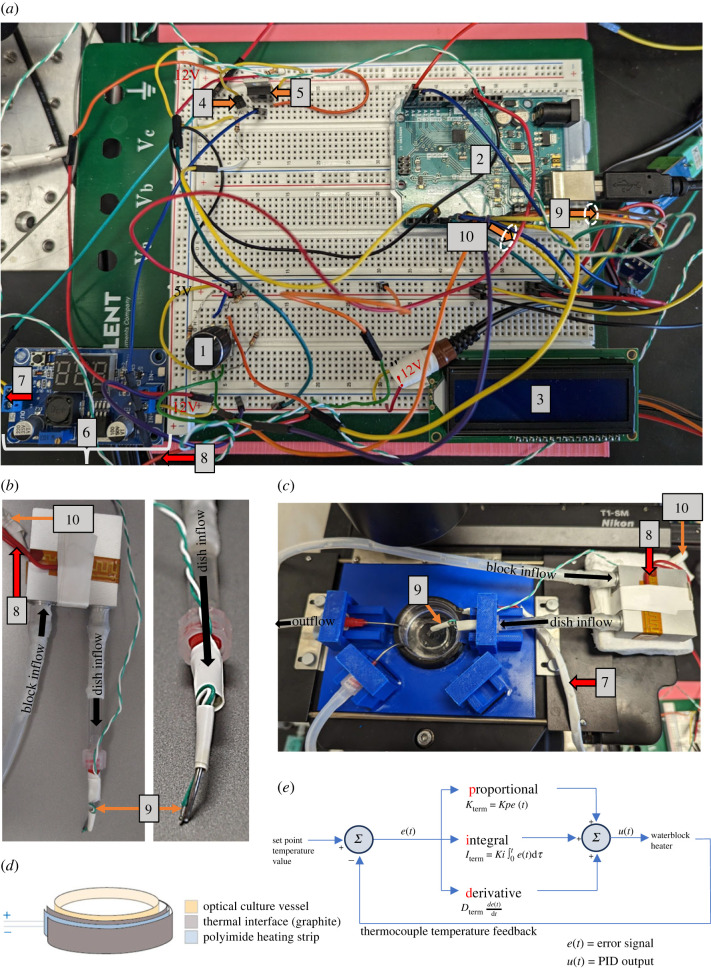
Thermal control components used in ThermoCyte. (*a*) Photograph of breadboard with key components labelled as per figure 1*c*: (1) Rotary encoder, (2) Arduino UNO REV 3, (3) I2C 16 × 2 liquid crystal display (LCD), (4) bipolar junction transistor (BJT), (5) metal oxide semiconductor field-effect transistor (MOSFET), (6) DC-DC step-down converter/voltage regulator, (7) wires to 12 V polyimide resistive heating strip glued to graphite thermal interface pad, Petri dish, (8) wires to 12 V polyimide resistive heating strip adhered to waterblock, (9) connections to K-type thermocouple at dish inlet (non-PID controlled), (10) connections to K-type thermocouple on undersurface of waterblock heating strip (PID-controlled). (*b*) Photograph of thermal control components: (8) waterblock polyimide heating strip, (9) thermocouple to dish, (10) thermocouple to waterblock heating strip. Direction of aCSF flow labelled with black arrows. (*c*) Photograph of stage-top set-up: (7) wires to 12 V polyimide resistive heating strip glued to graphite thermal interface pad, Petri dish, (8) wires to 12 V polyimide resistive heating strip adhered to waterblock, (9) connections to K-type thermocouple at dish inlet (non-PID controlled), (10) connections to K-type thermocouple on undersurface of waterblock heating strip (PID-controlled). (*d*) The stage-top used in this study was adapted to house a 13 mm optical Petri dish with glass bottom. A flexible thermal interface sheet of graphite (0.1 mm thick, 700 W mK^−1^) was layered on either side of the polyimdie heating strip, which was then affixed to the inside of a three-dimensional-printed partial ring, In this way, 13 mm Petri dishes could be snugly fit within the enclosure as needed. (*e*) A set-point temperature value is set using Arduino. This is summed with feedback from the waterblock thermocouple, and an error signal, *e*(*t*), generated. Proportional (*P*_term_), integral (*I*_term_) and derivative gain (*D*_term_) is calculated and summed, and output *u*(*t*) will be generated to manipulate the waterblock heating element. *P*_term_ =Kpe (t), proportional term, generates control action proportional to error; *I*_term_ $=Ki\int_{0}^{t}e\;(t)\;d\tau$, integral term, accumulates error over time; *D*_term_ = de(t)/dt, derivative term, generates control action proportional to rate of change of error.

In pursuit of an affordable yet effective solution for controlling the temperature of live-cell culture on a microscope stage-top, we developed ThermoCyte, using primarily off-the-shelf components. The principal components of our design included an Arduino UNO microcontroller, two polyimide resistive heating strips, and various electronic components dedicated to temperature control (figure 1*a*). A three-dimensional printer was employed to fabricate a customized microscope stage-top capable of housing our Petri dish, heater and tubing (figure 1*b*). All components were integrated into the temperature control circuit using a solderless breadboard (figure 1c and figure 2*a*). When operational, the system heats circulating solution at two points, in each case through a resistive polyimide heating strip. First, the anodized aluminium block is heated in line with temperature parameters set by the user (figure 2*b*). Then, the optical vessel itself is heated via a second polyimide strip shaped along the vessel's periphery within a three-dimensional-printed enclosure (figure 2*c,d*). This second strip runs at a constant input in order to counter conductive and convective heat loss to the environment stabilizing the temperature within the optical vessel and limiting temperature gradients across the vessel. Our objective was to ensure stable and reliable thermal dynamics, hence, a proportional, integral, derivative (PID) algorithm was implemented to govern the ‘process variable’—temperature in this case (figure 2*e*).

**Figure 3.**
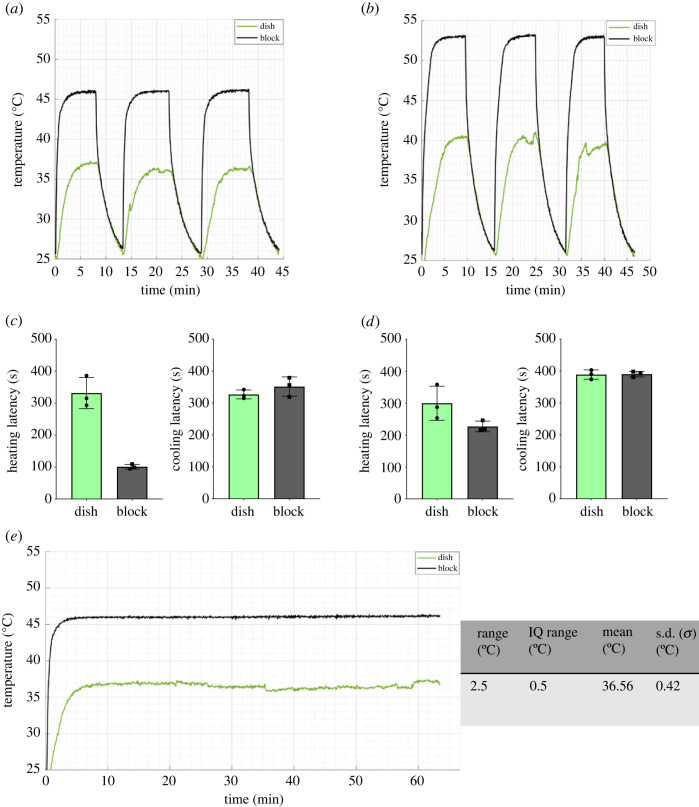
Thermal characteristics of heating system. (*a,b*) Artificial CSF (aCSF) was continuously circulated through the system at a rate of 2 ml min^−1^. A temperature set point for the heated waterblock was set (37°C or 40°C), and a log of temperature values from two thermocouples, one positioned at the Petri dish flow inlet (green) and one on the waterblock heating strip (black), is presented. Measurements were taken over three heating/cooling cycles from ambient temperature (approx. 25°C) to the chosen set-point temperature, and back to ambient temperature. (*c,d*) Graphs show heating and cooling latency values (s) for each thermal cycle, at set points of 37°C (*c*) and 40°C (*d*). Data are mean ± s.d., *n* = 1 independent replicate, *n* = 3 thermal cycles. (*e*) Artificial CSF (aCSF) was continuously circulated through the system at a rate of 2 ml min^−1^ for 1 hour. Graphs show temperature log from waterblock and dish thermocouples during a 60 min heating trial with set point 37°C. Adjacent table shows temperature range, interquartile (IQ) range, mean and standard deviation (*σ*). *n* = 1 independent replicate.

Following the completion of our design, we quantified the thermal performance of the system by conducting a series of heating and cooling trials. These trials logged system latency in transitioning from ambient temperature (approx. 20°C) to a predetermined set point, and vice versa, across three consecutive thermal cycles at an aCSF flow rate of 2 ml min^−1^ (figure 3*a*,*b*). At a set point of 37°C, the average heating and cooling latency of the Petri dish was 5.50 and 5.44 min, respectively. The waterblock heater exhibited a mean heating and cooling latency of 1.68 and 5.85 min (figure 3*c*). When the set point was raised to 40°C, the Petri dish showed an average heating and cooling latency of 4.98 and 6.48 min, respectively. The waterblock heater displayed a mean heating and cooling latency of 3.77 and 6.5 min (figure 3*d*). Moreover, we conducted an extended 1 h heating trial at 37°C without thermal cycling. During this trial, once the system initially reached the desired set point, the temperature fluctuation was within a 2.5°C range (minimum temperature, 35.25; maximum temperature, 37.75). The mean temperature recorded during this period was 36.56°C, with a standard deviation (s.d.) of 0.42°C (figure 3*e*). In our hands, evaporative loss was minimal. We recorded losses of 0.27% volume per hour, when imaging for 3 h and with a circulating superfusion volume of approximately 150 ml. However, solution replenishment may be necessary where smaller circulating volumes or longer experimental periods are required.

**Figure 4.**
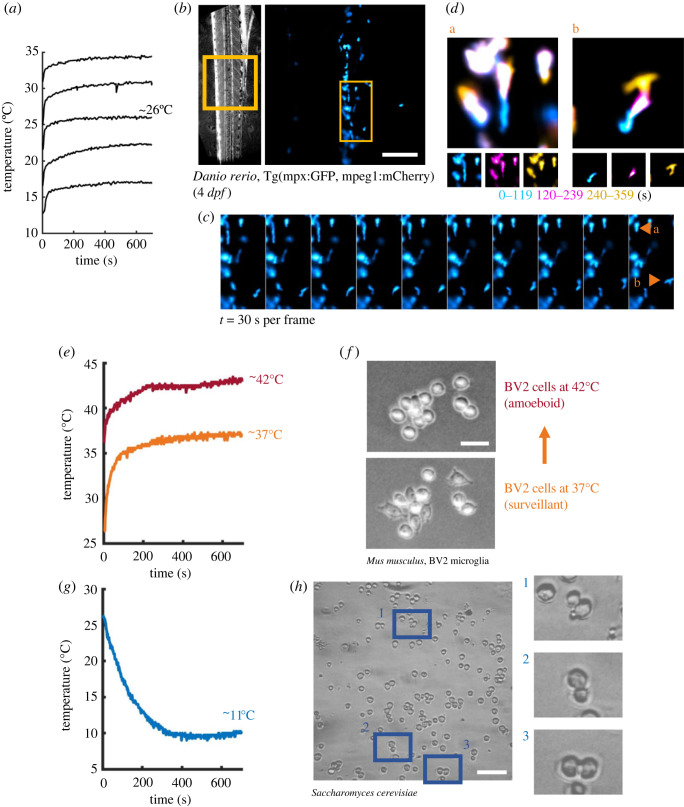
ThermoCyte permits multiple use cases across a variety of model organisms. (*a*) Increasingly high seek temperatures were set using the ThermoCyte interface and held for approximately 10 min, starting at 16°C and increasing to 22°C, 26°C, 30°C and finally 35°C. Solution was pre-cooled in ice to allow for temperature control below room temperature. (*b*) Transgenic zebrafish larvae (*D. rerio*, Tg(mpx:GFP, mpeg1:mCherry) at 4 days post-fertilization) were anaesthetized in tricaine and imaged at 26°C (similar to some tropical freshwater environments). Region of interest shown in brightfield image (*left*) was acquired using fluorescence microscopy (*right*) to observe GFP-positive neutrophils. Scale, 50 µm. (*c*) Montage of GFP-positive neutrophil dynamics in tail fin. Each frame shows a projection of a 30 s interval. (*d*) Details of two neutrophils from (*c*) with colour-coded timelapse projections elapsing 120 s. (E) BV2 microglia were observed at physiological temperature for 30 min (lower trace, showing initial stabilization over a 10 min interval) before ThermoCyte was used to increase temperature to 42°C. Microglia were imaged for a further 30 min. (*f*) Simulation of fever induces a morphological transition in BV2 microglia. Scale, 20 µm. (*g*) For imaging of *Saccharomyces cerevisiae* yeast cells, pre-cooled solution was circulated and heated to 30°C to activate cell cycling, before ThermoCyte was set to 10°C to facilitate rapid cooling, cell cycle arrest and imaging of various cell cycle stages (budding). (*h*) Brightfield images of *S. cerevisiae* yeast cells, with details provided for three cells at different stages of budding. Scale, 20 µm.

In order to demonstrate a wide variety of potential use cases for ThermoCyte, we applied heating control across a number of pilot experiments looking at different model organisms. Using pre-cooled solutions immersed in ice, we set seek temperatures of 16°C, 22°C, 26°C, 30°C and 35°C, ramping to the next highest temperature after a 10 min interval (figure 4*a*). Stable temperature control was achieved within 2–3 min. Once we had confirmed stability of various temperatures, we then performed pilot imaging of zebrafish larvae (*Danio rerio*, 4 days post-fertilization), simulating a tropical freshwater temperature of 26°C (figure 4*b*). Transgenic *D. rerio* expressing GFP in neutrophils were imaged, with neutrophils exhibiting surveillant behaviour within the tail fin (figure 4*c,d*). Next, we cultured BV2 microglia, a widely used mouse cerebellar microglial cell line [24–27], and performed imaging at physiological temperature, 37°C, for 30 min (figure 4*e*). Microglia at this temperature exhibit typical ramified morphology and localized surveillant motions. However, upon increasing the temperature to 42°C, to simulate a fever environment, localized motions decreased and microglia transitioned into more amoeboid morphologies, akin to inflammatory transitions (figure 4*f*). For the final pilot, we rehydrated desiccated *Saccharomyces cerevisiae* yeast in 0.9% saline solution with 200 mM sucrose, and heated the suspension to 30°C using ThermoCyte. A low peristaltic flow rate allowed for a number of yeast cells to passively rest of the optical surface for imaging, at which point budding could be observed. At this point, the inflow solution was switched for an ice-cold solution and the seek temperature was set to 10°C (figure 4*g*). Rapid cooling of the yeast led to cell-cycle arrest which facilitated imaging of S. *cerevisiae* at various stages of budding (figure 4*h*).

### 3.2. Biological validation of the custom temperature control system

**Figure 5.**
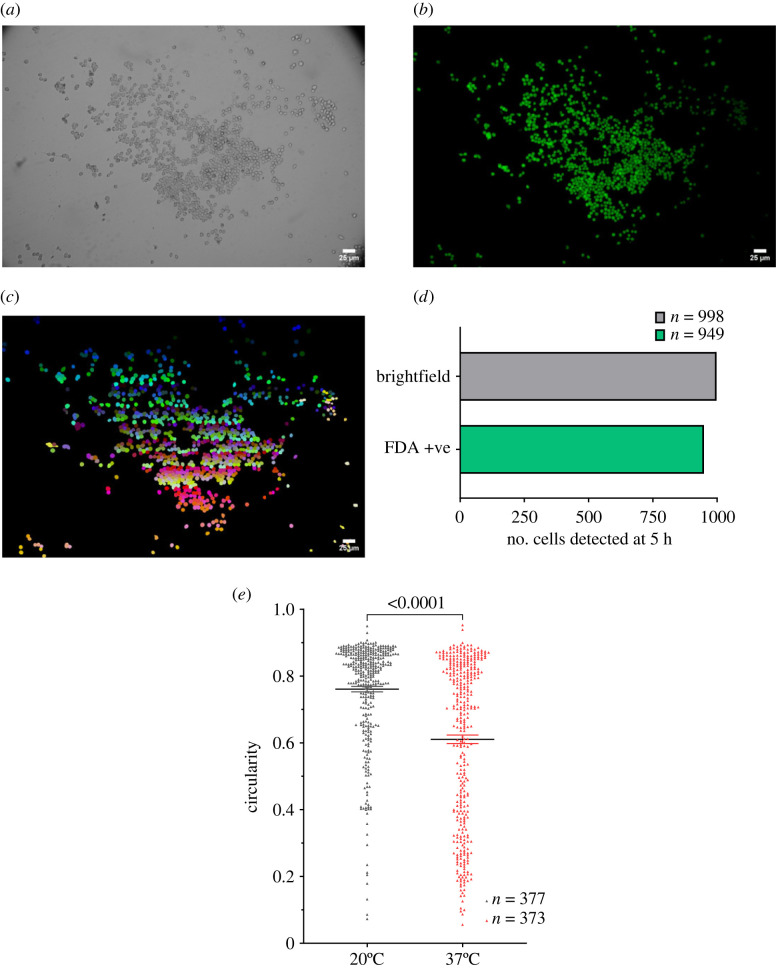
Cellular viability and morphology. (*a*) 10 000 BV2 microglia were cultured in a glass-bottomed Petri dish coated with 0.01% poly-L-lysine, in cDMEM, and incubated at 37°C, 5% CO_2_ for 3 days. Cells were then transferred into the stage-top heating system. Brightfield images were taken at 37°C. (*b*) Cells were incubated with fluorescein diacetate (FDA, 8 µg ml^−1^) on the microscope stage for 10 min in aCSF (no flow). Flow was then re-established, and FDA washed out for 5 min. Fluorescent images were taken using a custom-modified, fluorescence-enabled Nikon TS100 microscope. (*c*) Image showing boundaries of cells in images *a* and *b*. Boundary coordinates were determined by semi-automated object segmentation using the Python package ‘Cellpose’ and the ‘cyto2’ algorithm. (*d*) Graph shows the number of unstained and FDA^+^ cells using the above methods after 5 h at 37°C in the system. *n* = 1 independent replicate, *n* = 998 unstained cells, *n* = 949 FDA^+^ cells (95.1% total detected cells). (*e*) 10 000 BV2 microglia were cultured in a glass-bottomed Petri dish treated with poly-L-lysine, in complete DMEM, and incubated at 37°C, 5% CO_2_ for 3 days. Cells were loaded with Fluo4-AM calcium-dependent dye in aCSF for 1 h. Cells were then transferred into the stage-top heating system at 20°C or 37°C. Flow was established for 5 min to wash out residual dye. Cells were imaged using a custom-modified, fluorescence-enabled Nikon TS100 microscope. Cell boundary coordinates were determined by semi-automated object segmentation using the Python package ‘Cellpose’ and the ‘cyto2’ algorithm. Cellular morphology was measured using the ‘shape descriptors—circularity’ measurement feature in the ‘FIJI’ distribution of ‘ImageJ’ software. Graph shows circularity measurements of cells at 20°C and 37°C. Data represents mean ± s.e.m., *p* < 0.0001 pooled from *n* = 3 independent experiments. Statistical analysis was performed using the Mann–Whitney *U* test, *p* < 0.0001.

Having established stable thermal dynamics in our system applicable to multiple model organisms, we sought to apply heating control within our experimental set-ups requiring live-cell imaging. To test the biocompatibility of our system, we used BV2 microglia to quantify the number of viable cells remaining at the end of a 3 h imaging trial at 37°C. BV2 cells were incubated with the viability probe fluorescein diacetate (FDA, 8 µg ml^−1^) stain while being imaged on our stage-top. We assessed the proportion of fluorescein-positive cells at the end of the imaging session as an indicator of gross cellular viability [28–31]. We sampled a large region of interest, identifying 998 cells via brightfield microscopy (figure 5*a,b*). Out of these, 949 (95.1%) were FDA^+^, corroborating the biocompatibility of ThermoCyte (figure 5*c,d*). In addition, we performed a morphological analysis of BV2 microglia in our system. Microglial morphology exists on a broad spectrum, and the position on that spectrum (i.e. ‘resting’ versus ‘activated’, ‘M2-like’ versus ‘M1-like’) is correlated with cellular stress and immune phenotype [13,14,16]. Thus, assessing cell morphology provided a supplementary marker of ThermoCyte's cytocompatibility. The morphology of each cell was quantified using the parameter ‘circularity’, which is expressed as $4\times \Pi \times (\mathrm{area}÷{\mathrm{perimeter}}^{2})$. A value of 1.0 denotes a perfect circle, and values less than 1.0 indicate progressively less circular morphologies. Cells imaged at 20°C had a mean circularity of 0.761, in contrast to those imaged at 37°C, which had a mean circularity of 0.610, indicating a temperature-dependent shift from amoeboid to more ramified, quiescent phenotypes (figure 5*e*, ***p < 0.001).

### 3.3. ThermoCyte enables live-cell calcium imaging at physiological temperature

**Figure 6.**
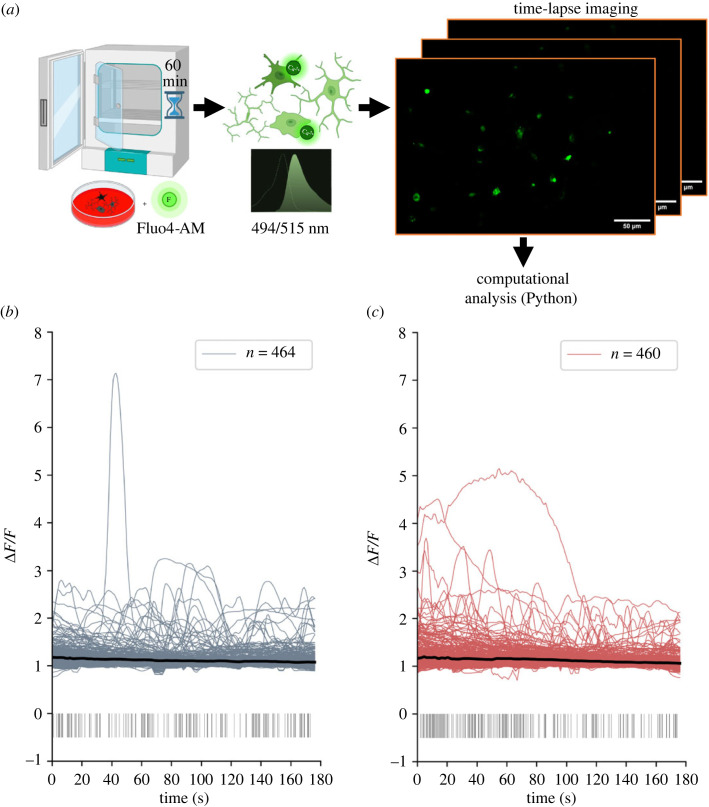
Heating system permits analysis of calcium signalling activity at physiological temperature. (*a*) Schematic showing calcium imaging protocol in BV2 microglia. 10 000 BV2 microglia were cultured in a glass-bottomed Petri dish treated with poly-L-lysine, in complete DMEM, and incubated at 37°C, 5% CO_2_ for 3 days. Cells were loaded with Fluo4-AM calcium-dependent dye in aCSF for 1 h. Cells were then transferred into ThermoCyte at 20°C or 37°C. Flow was established for 5 min to wash out residual dye. Cells were imaged over 3 min trials using a custom-modified, fluorescence-enabled Nikon TS100 microscope. Time-lapse images were acquired, and cell boundary coordinates were determined by semi-automated object segmentation using the Python package ‘Cellpose’ and the ‘cyto2’ algorithm. Mean pixel intensity for each cell at 20°C (*b*) or 37°C (*c*) over 3 min was extracted using the ‘FIJI’ distribution of ‘ImageJ’ software. Pixel intensity traces were de-noised, normalized to change in fluorescence/baseline fluorescence (ΔF/F), and calcium signalling events (peaks) were detected with code written using the ‘Anaconda’ distribution of Python. Graphs show line plot of signal traces of each cell over 3 min with the average waveform of call cells in black. Calcium signalling peaks greater than or equal to 2 s width and greater than or equal to 0.5 ΔF/F height are detected and shown as vertical lines below main plot. Legend shows *n* = number of cells analysed. Data pooled from *n* = 3 independent experiments. Some parts of figures created with Biorender.com.

**Figure 7.**
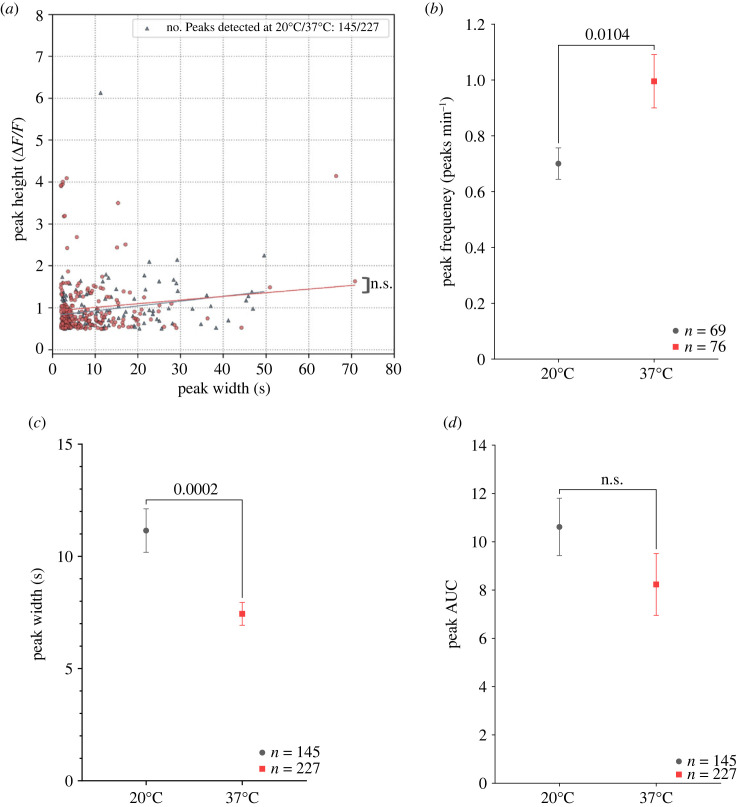
Kinetics of baseline calcium signalling may be temperature dependent in BV2 microglia. 10 000 BV2 microglia were cultured in a glass-bottomed Petri dish treated with poly-L-lysine, in complete DMEM, and incubated at 37°C, 5% CO_2_ for 3 days. Cells were loaded with Fluo4-AM calcium-dependent dye in aCSF for 1 h. Cells were then transferred into ThermoCyte at 20°C or 37°C. Flow was established for 5 min to wash out residual dye. Cells were imaged over 3 min trials using a custom-modified, fluorescence-enabled Nikon TS100 microscope. Time-lapse images were acquired, and cell boundary coordinates were determined by semi-automated object segmentation using the Python package ‘Cellpose’ and the ‘cyto2’ algorithm. Mean pixel intensity for each cell at 20°C or 37°C over 3 min was extracted using the ‘FIJI’ distribution of ‘ImageJ’ software. Pixel intensity traces were de-noised, normalized to change in fluorescence/baseline fluorescence (Δ*F*/*F*), and calcium signalling events (peaks) were detected with code written using the ‘Anaconda’ distribution of Python. (*a*) XY scatter plots of peak height (ΔF/F) versus peak width (s) at 20°C (grey) and 37°C (red) respectively. Statistical analysis was performed by comparing the slopes of the best-fit lines generated by a least squares linear regression model. (*b*) Graph shows peak frequency of cells at 20°C and 37°C (mean ± s.e.m.; statistical analysis was performed using an unpaired *t*-test, *p* = 0.0104). (*c*) Graph shows peak width 20°C and 37°C (mean ± s.e.m.). Significance was calculated using an unpaired-*t* test, *p* = 0.0002. Data are *n* = 3 independent replicates. (*d*) Graph shows mean area-under-curve (AUC) value for calcium signalling events (peaks) at 20°C and 37°C (mean ± s.e.m.). Statistical analysis was performed using an unpaired *t*-test, *p* = 0.2013. Data are *n* = 3 independent replicates.

To further explore use cases for ThermoCyte and demonstrate how temperature control impacts biological sampling, we applied ThermoCyte to more complex, relevant biological imaging assays; specifically, live-cell calcium imaging. We established a protocol for calcium imaging in BV2 microglia using Fluo4-AM, a membrane-permeant calcium-dependent fluorescence-based dye [32,33] (figure 6*a*). Baseline imaging trials at 20°C and 37°C were successfully conducted in our system, and calcium signals from each cell, in the form of relative fluorescence intensity changes (Δ*F*/*F*), were analysed using custom Python code (figure 6*b,c*). Subsequently, we explored whether the selected temperature during imaging impacted the calcium signalling kinetics observed. In unstimulated cells, the distribution of constitutive calcium fluctuations, as assessed through the peak parameters ‘height’ versus ‘width’, showed no significant change between the two temperatures (figure 7*a*, slopes compared using a least-squares linear regression model). We then classified cells into ‘inactive’ and ‘active’ populations. Active cells were defined as having at least one peak of 0.5 ΔF/F height and 2 s width detected by our algorithm. Increasing temperature from 20°C to 37°C significantly increased peak frequency in active cells from 0.7 to 0.99 peaks min^−1^ (figure 7*b*, unpaired *t*-test, *p = 0.0104). Concurrently, peak width was also significantly reduced by an increase in temperature, from 11.14 s at 20°C to 7.14 s at 37°C. (Figure 7*c*). However, there was no observed difference between the area-under-curve (AUC) values of each individual peak (figure 7*d*). Considering these findings, we turned our focus towards understanding how these cells might respond to adenosine triphosphate (ATP), a potent inflammatory mediator and activator of purinergic receptors [32,34,35], at each temperature.

### 3.4. BV2 calcium signalling kinetics in response to adenosine triphosphate is influenced by imaging temperature in ThermoCyte

**Figure 8.**
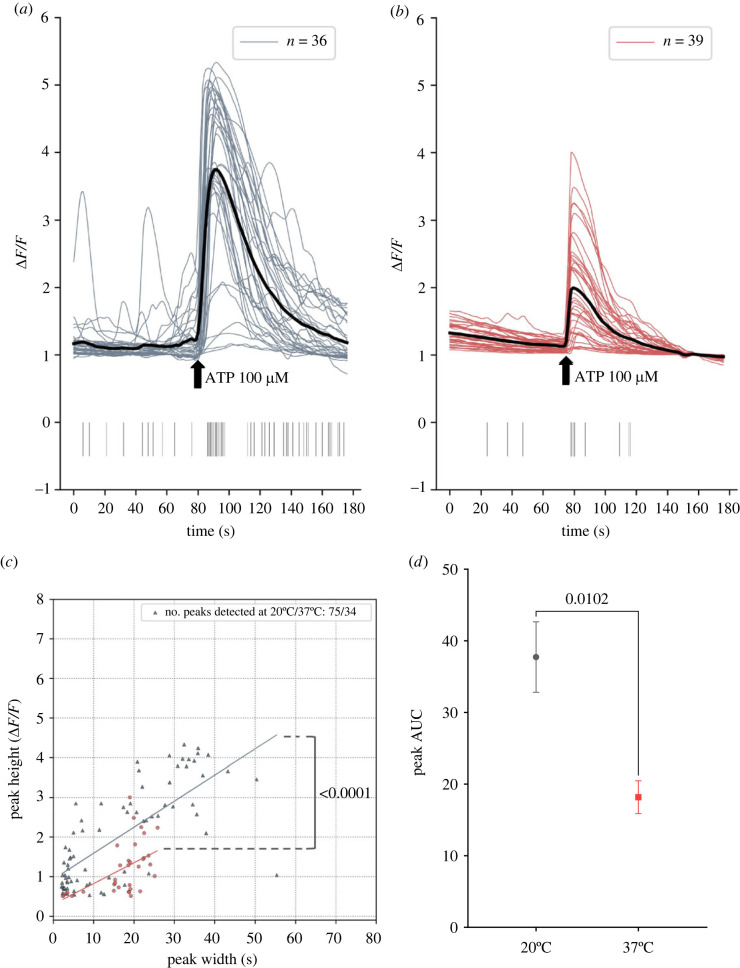
Kinetics of calcium signalling in response to adenosine triphosphate (ATP) is temperature dependent in BV2 microglia. 10 000 BV2 microglia were cultured in a glass-bottomed Petri dish coated with poly-L-lysine, in complete DMEM, and incubated at 37°C, 5% CO_2_ for 3 days. Cells were loaded with Fluo4-AM calcium-dependent dye in aCSF for 1 h. Cells were then transferred into the stage-top heating system at 20°C or 37°C. Flow was established for 5 min to wash out residual dye. Cells were imaged over 3 min trials using a custom-modified, fluorescence-enabled Nikon TS100 microscope. ATP (100 µM) was added to the petri dish at 80 seconds, and the response was recorded with flow disabled. Time-lapse images were acquired, and cell boundary coordinates were determined by semi-automated object segmentation using the Python package ‘Cellpose’ and the ‘cyto2’ algorithm. Mean pixel intensity for each cell at 20°C (*a*) or 37°C (*b*) over 3 min was extracted using the ‘FIJI’ distribution of ‘ImageJ’ software. Pixel intensity traces were de-noised, normalized to change in fluorescence/baseline fluorescence (ΔF/F), and calcium signalling events (peaks) were detected with code written using the ‘Anaconda’ distribution of Python. Graphs show line plot of signal traces of each cell over 3 min with the average waveform of call cells in black. Addition of ATP is denoted by a black arrow. Calcium signalling peaks greater than or equal to 2 s width and greater than or equal to 0.5 ΔF/F height are detected and shown as vertical lines below main plot. Legend shows *n* = number of cells analysed (*c*) XY scatter plots of peak height (ΔF/F) versus peak width (s) at 20°C (grey) and 37°C (red). Legend shows the number of peaks analysed. Lines of best fit were generated using a least-squares linear regression. Statistical analysis was performed using an analysis of covariance (ANCOVA) to compare intercepts, *p* < 0.0001. Data represents *n* = 1 independent replicate.

After establishing a baseline imaging protocol, BV2 microglia were stimulated with 100 µM ATP, a relevant extracellular damage-associated molecular pattern (DAMP) [34,36], after 80 s of baseline imaging. The resulting signalling kinetics, observed at 20°C (figure 8*a*), and 37°C (figure 8*b*) showed significant differences. Each signalling event (peak) was analysed using the parameters ‘height’ versus ‘width’, employing the same criteria as prior experiments: 0.5 ΔF/F height and 2 s width. Notably, the distribution of peaks at 37°C was significantly shifted downwards in comparison with 20°C. This was deduced by constructing a least squares regression line and conducting an analysis of covariance (ANCOVA) (figure 8*c*, ****p < 0.0001). Finally, mean area-under-curve (AUC) of each detected peak was also measured, revealing significantly higher AUC values for peaks at 20°C in contrast to those at 37°C (figure 8*d*, ***p = 0.0102). After this extensive activity analysis of microglia in ThermoCyte, we also investigated the feasibility of using a streamlined version of the system's hardware, devoid of aCSF superfusion. This simplified approach was primarily done to test ThermoCyte's modular capabilities and may appeal to researchers who are not in need of a superfusion set-up.

### 3.5. Cellular motility in GFP^+^ murine-induced pluripotent stem cell (miPSC)-derived microglia

**Figure 9.**
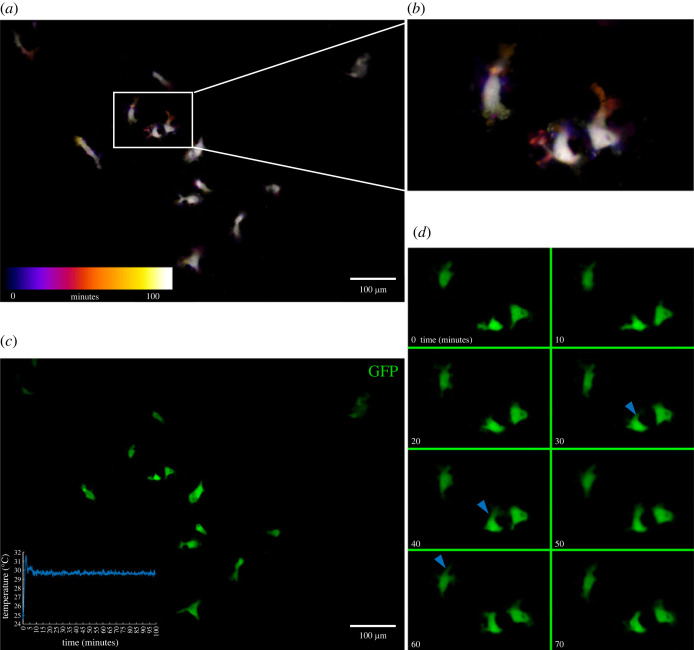
Cellular motility in miPSC-derived GFP^+^ microglia is observed using ThermoCyte. (*a*) 10 000 mature GFP^+^ microglia (§2.8) were seeded onto a glass-bottomed Petri dish coated with 20 µg ml^−1^ fibronectin, in complete GMEM, and incubated at 37°C, 5% CO_2_ for 3 days. Cells were then transferred into the stage-top heating system at 30°C for 100 minutes, with only the Petri dish heater activated (i.e. no waterblock heating element or aCSF flow). Images were acquired at 3 min intervals using a custom-modified, fluorescence-enabled Nikon TS100 microscope. Cellular motility was analysed using the ‘temporal heat map’ function in the ‘FIJI’ distribution of ‘ImageJ’ software. (*b*) Cropped-in view of temporal heat map in image *a*. (*c*) Fluorescent micrograph of microglia and associated temperature log over a 100 min trial. (*d*) Collage showing microglial motility over 70 min of a 100 min trial at 30°C.

To confirm the modular capabilities of ThermoCyte, we sought to perform basic live-cell imaging in a stripped-down configuration, this time using primary-derived (miPSC, CX_3_CR1^eGFP/+^CCR2^RFP/+^) microglia. As these cells are intrinsically fluorescent, they are well-suited to analysis of cellular motility using fluorescent time lapse imaging. Cells were imaged using the previously described modified epifluorescence microscope. However, only the Petri dish heating element of ThermoCyte was employed in this set-up, eliminating the need for a waterblock, its associated heating element, or aCSF superfusion. Cellular motility was visualized using a temporal heat map (figure 9*a,b*). Microglia remained motile and retained baseline morphology throughout a 100 min imaging trial at 30°C (figure 9*c,d*).

## 4. Discussion

Here we have developed an effective heating system which stably maintains a temperature set point on the microscope stage. Importantly, one of the major advantages of our system is the total cost. The price of our electrical and heating components is around €200 after VAT, excluding shipping (table 1). Importantly, all components used are easily sourced, off-the-shelf components. For comparison, there are numerous commercially available in-line heating solutions available at the time of publication, with advertised prices ranging up to approximately €3800 [37]. Although these systems are accurate and featureful, we see ThermoCyte as a simple, cost-effective and functional alternative for heated live-cell imaging on the microscope stage.

**Table 1.** Listed materials for construction of ThermoCyte with associated price guides.

cost of materials (electronics and heating components)	
ITEM	PRICE (€)
Arduino UNO rev 3.0	22.80
solderless breadboard (large)	56.65
jumper wires	12.99
resistors (5)	1.44
DC-DC stepdown converter	7.79
metal oxide semiconductor field-effect transistor (MOSFET)	1.67
bipolar junction transistor	0.46
rotary encoder	2.12
Arduino I2C liquid crystal display (LCD)	13.47
waterblock	10.95
polyimide heating strip × 2	6.54
K-type thermocouple (1 M) × 2	22.18
MAX-6675 thermocouple amplifier × 2	19.04
three-dimensional printing costs (excl. printer & filament)	2.00
graphite thermal interface sheet	20.90
TOTAL COST	201.00

ThermoCyte is adaptable, and all components can be changed with some simple coding experience. As evidenced by our experiment using miPSC-derived microglia (figure 9), running the system using only the Petri dish heating element at a lower temperature of 30°C still permitted clear cellular motility and maintenance of baseline cell morphology. ThermoCyte's modular capabilities are a strong advantage for the price. A major future adaptation could, for example, include a Peltier device (thermoelectric heater or cooling module) being installed in place of the polyimide heater on the waterblock, in a ‘plug and play’ fashion. This would require little modification to the Arduino code and would allow the imaging medium to be actively cooled or heated, facilitating studies into cellular hypothermia and fever. Individual researchers may identify different applications of our system and will be able to easily modify the original hardware to suit their experimental requirements.

Critically, ThermoCyte is compatible with a widely used cell line, murine BV2 microglia, and cells derived from a primary source, miPSC-derived microglia. It permits imaging over extended trials (up to 5 h long tested), with FDA-staining being strongly positive in the majority (95.1%) of BV2 microglia, indicating good biocompatibility. An imaging trial of 5 h duration is ample to allow for complex calcium imaging experiments, such as those requiring the application of receptor agonists and inhibitors (such as ATP in this case).

ThermoCyte has highly stable thermal control, as evidenced by steady heating and cooling latency values for the Petri dish and waterblock heating element, across three thermal cycles (figure 4*a–d*). Furthermore, a 1 h extended trial demonstrated a narrow temperature range (2.5°C), with low standard deviation (0.42°C) and interquartile range (0.5°C) at set-point temperature. Together these values confirm ThermoCyte's thermal stability. Additionally, the easily tunable temperature set point will enable its use in diverse models. Examples may include studies performed at physiological temperature, studies investigating cellular response to fever, and experiments necessitating rapid temperature cycling.

Critically, the behaviour of cells in ThermoCyte changes depending on the imaging temperature chosen. This was initially evidenced by our studies into cellular circularity, showcasing a significant temperature-dependent change in cellular morphology. Cells had higher circularity at 20°C, and lower circularity at 37°C (figure 5*e*). This indicates a trend towards a more amoeboid, or round, population at 20°C, but a trend towards a more ramified, or less round, population at 37°C. Ramification is known to correlate with ‘surveilling’ or ‘resting’ immune phenotype, as compared with the ‘activated’ phenotype that is associated with an amoeboid morphology [13–16]. This suggests that ThermoCyte may indeed reduce levels of microglial activation, and possibly even cellular stress, at 37°C. However, the exact effect of temperature on microglial state dynamics is out of the scope of this paper. A comprehensive summary of microglial morphology can be found in Leyh *et al.* [16].

Another significant advantage of ThermoCyte is best evidenced by calcium signalling activity in BV2 microglia. At baseline, calcium signalling kinetics are lower in amplitude, and exhibit a shorter duration at 37°C, in comparison with 20°C (figure 7*b,c*). Evidence of altered kinetics may also be present in baseline peak AUC measurements (figure 7*d*), although this was not statistically significant. Importantly, changes in signalling kinetics were also observed in microglia stimulated by ATP, with calcium transients prolonged at 20°C as compared with 37°C (figure 8*a–c*). In addition, peak AUC values were significantly decreased at 37°C as compared with 20°C. It is likely that the observed changes in calcium activity are being driven by temperature-dependent changes in the activity of the cells, where calcium dynamics are altered through changes in the steady-state of cytosolic buffering, intracellular storage and extracellular uptake [38–40]. It also important to note, however, that Fluo4-AM exhibits temperature-dependent changes to its physical chemistry. Single-wavelength calcium-dependent indicators are known to exhibit increased fluorescent lifetimes, as well as increased peak (transient) amplitude, at cooler temperatures. This occurs for a variety of reasons, including changes to dissociation constant (K_d_) [41,42]. The chemistry of Fluo4-AM may contribute to our observed results; however, we believe this is a less likely explanation than temperature-dependent biological changes. Together, these data suggest that ThermoCyte enables accurate imaging and analysis of live-cell calcium signalling kinetics at physiological temperature. By this, one can ensure that inferences made about transient amplitude, duration and frequency are accurate, and that differences in these parameters are not masked by altered kinetics at ambient temperature.

Importantly, some limitations of our system should be acknowledged:

1. *Thermal gradients*. A significant limitation is the existence of a thermal gradient across the Petri dish. Dish temperature is measured at the hottest point in the dish, which is the centre point, under the inlet for aCSF flow. By nature of thermodynamics, the solution will variably cool before being removed by the outlet. By our measurements, this is approximately 2°C using both the circumferential Petri dish heating strip and the heated waterblock. This ‘dual heating’ method is effective as both aCSF entering, and peripheral Petri dish, are being directly heated. This small thermal gradient may be circumvented by heating under the Petri dish with a heated stage-top. However, this will reduce how much of the dish surface is visible and may restrict imaging. This choice will depend on specific researcher requirements. Though this is outside of the scope of our design described here, it can be easily incorporated using modifications to the printed stage, the electronic components and the Arduino implementation.
2. *Manual temperature control.* Our set-up in its current configuration lacks automation of temperature cycles. Currently, we can choose a temperature set point for the waterblock, and thus the dish, to remain at. For more complex temperature profiles or cascade control systems, additional editing of code will be required; however, this should be straightforward with some basic Arduino programming experience.
3. *Passive cooling.* Passive cooling latency is rather slow, which is expected due to hysteresis. However, this may be problematic for those who wish to employ rapid temperature cycles in the imaging dish. This, however, may again be circumvented by using a Peltier module on the waterblock, activated only to actively cool aCSF as required.
4. *Air trapping.* As is the case in most live-cell superfusion studies, care must be taken to avoid air trapping and bubble formation, particularly within the flow inlet circuit. If generated, air bubbles may obstruct tubing and cause the flow to reduce or cease. We have circumvented this problem with a bubble trap, constructed out of a sealed 20 ml syringe (figure 1*a*). Although bubble formation has rarely been problematic, this device has a theoretical gas volume limit. For this reason, care must be taken to avoid introducing air into the system during set-up, and it may be necessary to purge the system of air bubbles manually should this occur.

To circumvent many of these limitations, and further improve upon the system, there are many theoretical configurations that may be more appropriate for certain scientific applications. While beyond the scope of this paper, we suggest some potentially useful configurations:

*1. Gravity feed*. Using a peristaltic pump adds cost to the system. In addition to this, the nature of flow generated by these pumps is pulsatile. This variation in flow rate may not suit all research applications. Thus, replacing the peristaltic pump with a reservoir set to a certain height will improve upon the stability of flow rate. Flow rate can be measured manually, or approximately calculated using the following mathematical formulae: (A) hydrostatic pressure equation; p=ρgh; *p* is hydrostatic pressure (N m^−2^), *ρ* (rho) is liquid density (kg m^−3^), *g* is acceleration due to gravity (9.81 m s^−2^), *h* is height of liquid (m). (B) Hagen-Poiseuille equation; $Q=(\pi \times \Delta P\times r^{4})/(8\times \eta \times L)$; *Q* is flow rate (m^3^ s^−1^), Δ*P* is pressure difference between two ends of tubing (Pa), *r* is internal radius of tubing (m), η is dynamic viscosity of fluid (Pa s), *L* is length of tubing (m), *π* is pi (3.14159).
*2. Different culture vessels*. To create our stage-top Petri-dish adaptor, we used a basic Ender 3 Pro 3D printer. This design could be easily modified to support Petri dishes of different sizes and shapes, as well as other culture vessels altogether. Researchers will need to consider the thermal implications of increasing vessel size. Heating the imaging media directly via the aluminium waterblock should not be adversely affected. However, redesign of the indirect dish heating strip configuration may be required, if necessary.
*3. Objective heater.* Using the same principle as heating the Petri dish, one could use a polyimide heating strip to heat a dipping or objective lens directly, should it be required. This should take no more than an additional heating strip, 12 V power supply, thermocouple and amplifier, and PID control electronics as described.
*4. Environmental sensors.* To further improve on the system, researchers may interface additional sensors with the Arduino to achieve more optimal environmental control. Examples include dedicated pH, osmolarity, CO_2_ and humidity sensors. These components are off the shelf and should be easily integrated with the system as it currently stands.
*5. Programmable components.* It is possible to program Arduino to control optical components. Specifically, the automated control of the microscope shutter or LED driver and image capture mechanisms would prove to be a significant time saving measure and permit longer imaging trials. An important consideration with this method is the reduction and correction of focus drift, which may become problematic during longer imaging sessions. Further, the use of the Arduino will facilitate additional modular control of optomechanical elements, including motorized stage controllers which, in combination with autofocus routines, could achieve long-term image stability. Additionally, Arduino should be easily interfaceable with custom syringe pumps (e.g. stepper motor-based systems). This would permit automated stimulation of cells with agonists/inhibitors as required.

In conclusion, our system represents a simple, cost-effective and versatile method for basic live-cell imaging studies on the microscope stage-top, at physiological temperature. Although more advanced and featureful systems are available, these are significantly costlier and may be out of reach and impractical for many researchers. Prior efforts have been made to engineer inexpensive thermal devices for optical imaging. A notable example is the ‘ringcubator’, consisting of a custom-heated aluminium microscope stage-top [43], and our approach complements such a device. We have similarly chosen to heat around a Petri dish directly but using direct contact with a graphite thermal interface sheet, rather than a fabricated aluminium stage-top. Furthermore, our use of a heated anodized aluminium waterblock is a novel DIY approach to this problem. Additionally, major advancements in microcontroller technology have allowed us to use platforms like Arduino, which are widely known and taught worldwide. This will ensure that this project is globally accessible to those with and without electronics or coding knowledge. Owing to its modular, customizable and adaptable nature, we envision ThermoCyte as a catalyst which will inspire researchers to build, test and utilize their own systems to further explorations in the field of live-cell imaging.

## Data Availability

All research materials supporting the data described in the manuscript have been provided either within the main text itself or in the electronic supplementary material. All data have also been uploaded to the Dryad Digital Repository: https://doi.org/10.5061/dryad.2280gb5zd [44]. Supplementary material is available online [45].
